# The role of neutrophils and NETosis in lipopolysaccharide exacerbated asthmatic airway inflammation

**DOI:** 10.3389/fimmu.2025.1651085

**Published:** 2025-09-02

**Authors:** Qian Qian, Qianye Zhao, Yongji Qian, Huan Deng, Xiaoming Feng, Jiamin Zhang, Xia Huang, Yi Du, Deyu Zhao, Feng Liu

**Affiliations:** ^1^ Department of Respiratory Medicine, Children’s Hospital of Nanjing Medical University, Nanjing, China; ^2^ Department of Respiratory Medicine, Lianyungang Maternal and Child Health Hospital, Lianyungang, China; ^3^ Department of Traditional Chinese Medicine, Lianyungang Traditional Chinese Medicine Branch of Jiangsu Union Technical Institute, Lianyungang, China

**Keywords:** lipopolysaccharide, neutrophilic asthma, neutrophil extracellular traps, differentially expressed genes, airway inflammation

## Abstract

**Background:**

Lipopolysaccharides (LPS) are associated with the exacerbation of asthma, accompanied by an increased recruitment of neutrophils to the airway. The role of these neutrophils warrants thorough investigation.

**Methods:**

In this study, three genotypes of mice were utilized to establish an asthma model aggravated by LPS combined with ovalbumin (OVA). The bronchoalveolar lavage fluid (BALF) of mice was obtained to detect neutrophil-related inflammatory factors. Lung tissues were collected for staining, and neutrophils derived from bone marrow of mice were subjected to transcriptomic sequencing analysis.

**Results:**

Our findings revealed that, compared to eosinophilic asthma, Exacerbated asthma triggered by LPS combined with OVA showed more severe airway inflammation. Neutrophil-related markers like IL6, IL8, and neutrophil extracellular traps (NETs) were significantly elevated in this model. Inhibiting neutrophils production significantly improved airway inflammation and lung function. Analysis of differentially expressed genes (DEGs) in bone marrow neutrophils highlighted enrichment in the NETs pathway. Suppressing NETs yielded similar results to decreasing neutrophils.

**Conclusion:**

Our results indicate that NETs are involved in the pathogenesis of LPS exacerbated asthmatic airway inflammation, and targeting the NETosis function of neutrophils may represent an effective therapeutic approach.

## 1 Introduction

Globally, asthma affects over 300 million individuals and is responsible for the annual mortality of approximately 42,000 patients (1). Its prevalence increases by about 50% each decade (2). Asthma is clinically characterized by recurrent cough, shortness of breath, chest tightness, and airway hyperresponsiveness. The involvement of various inflammatory cells, airway structural cells, and their components underscores the complex pathogenic mechanisms underlying asthma. To enhance the understanding of asthma pathogenesis, four inflammatory phenotypes are classified based on the proportion of granulocytes present in induced sputum. Among them, neutrophilic asthma, a subtype of T2-low asthma, is frequently associated with severe disease and a poor response to inhaled corticosteroids (ICS) (3).

LPS, an essential component of the outer membrane of gram-negative bacteria, triggers a significant pro-inflammatory cascade that can culminate in acute lung injury. Whereas antigen-antibody complexes activate complement via the classical pathway, LPS is capable of initiating complement activation through multiple pathways (4). This activation generates anaphylatoxins (C3a and C5a) and membrane attack complexes (C5b-9), key mediators contributing to inflammatory tissue damage (5). Critically, excessive complement activation, characterized by elevated levels of C3a and C5a, may also exacerbate asthma pathology (5, 6).

LPS originating from the environment or respiratory tract bacterial infections can also interact with pattern recognition receptors in the airway, leading to exacerbation of asthma or asthma-like symptoms (7, 8). Upon exposure to LPS, allergic eosinophilic asthma progresses towards a neutrophilic phenotype, which is characterized by T-helper (Th)1 and Th17 responses (9). Numerous mouse studies have demonstrated that when LPS is employed as an adjuvant in the establishment of a mouse neutrophilic asthma model, the resultant airway inflammation and hyperresponsiveness are significantly aggravated compared to classic OVA or house dust mite (HDM) models, often accompanied by an obvious increase in airway neutrophils (3, 10, 11).

The increased infiltration of neutrophils in airway is an important pathological feature of this type of asthma, suggesting that neutrophils may play a pivotal role. Neutrophils, as is well-established, serve as a crucial component of innate immunity, rapidly migrating to sites of acute inflammation during infection or injury to exert their protective functions. Recent research increasingly indicates that neutrophils are implicated in the progression of chronic inflammatory diseases, including asthma (12). Compared with the healthy control group, the peripheral blood neutrophil count of inpatients with asthma was significantly higher (13). Similar results were found in pediatric patients with severe asthma attacks who were admitted to the intensive care unit (ICU). Notably, however, the proportion of eosinophils did not show a corresponding increase (14). Neutrophils isolated from the BALF of children with severe asthma exhibited heightened activation and extended survival compared to those from healthy controls (15). In a mouse model of asthma exacerbation induced by rhinovirus or LPS, neutrophils were rapidly recruited to the lungs, peaking at 24 hours, which preceded the type 2 inflammatory response (16, 17). Consequently, the precise mechanisms underlying neutrophils in neutrophilic airway inflammation warrant further investigation.

Here, we employed three genotypes of mice and induced neutrophilic airway inflammatory asthma by using LPS and OVA. Our findings indicate that the suppression of neutrophils and NETosis significantly mitigates the aforementioned airway inflammation.

## 2 Methods

### 2.1 Animals

C57BL/6J female mice (age, 6–8 weeks, body weight, 18–20 g) were purchased from the animal core facility of Nanjing Medical University. Colony-stimulating factor 3 deficient (*Csf3* ^-/-^) and peptidyl arginine deiminase 4 deficient (*Padi4* ^-/-^) C57BL/6J female mice were purchased from Cyagen Biosciences (Suzhou, China). Genotypes were identified by PCR analysis of tail DNA (**Supplementary Figures 1A, B**). These animals were housed in specific pathogen–free (SPF) conditions,12/12 h controlled light conditions with ad libitum access to water and food. All mice experiments were performed with approval from the Institutional Animal Care and Use Committee, Nanjing Medical University (reference number: IACUC-2208009). We guarantee that all experimental procedures were carried out in accordance with the guidelines established by the Institutional Animal Care and Use Committee, Nanjing Medical University. These experimental methods complied with Helsinki Declaration, and every effort was made to minimize suffering.

### 2.2 Animal models of asthma

The mouse model was carried out with reference to the previous literature with slight modification (3, 18). The mice were randomly assigned to four groups: the phosphate buffered saline (PBS) group, the LPS group, the OVA group, and the LPS + OVA group. mice were intraperitoneally (i.p.) injected with 100μg OVA (grade V, Sigma-Aldrich, USA) and 50μl aluminum hydroxide gel (Thermo Fisher Scientific, USA) dissolved in 100μl PBS or 50μl aluminum hydroxide gel dissolved in 100μl PBS in non-OVA groups during the sensitization phase on days 0 and 7. From days 14 to 16, these mice were challenged by intratracheally (i.t.) instillation of OVA (25μg OVA dissolved in 50μl PBS) or PBS in non-OVA groups. Then, sacrificed the mice on day 17. In LPS and LPS+OVA groups,10μg LPS from Escherichia coli 0111: B4 (Sigma-Aldrich, USA) dissolved in 50μl of PBS was administered by intratracheal instillation on days 0 and 7.LPS was replaced by PBS in non-LPS groups. mice were lightly anesthetized with pentobarbital sodium when intratracheal intubation for drug administration was required.

### 2.3 Bronchoalveolar lavage and cell count

Tracheas were cannulated and the right lung was lavaged slowly 3 times with 0.5ml PBS following the left lung ligation. The recovery rate of the BALF was greater than 60%. Each fluid was centrifuged and the supernatant was rapidly frozen at -80°C. The cells in BALF were resuspended in PBS and centrifuged in a cytocentrifuge, then stained with Wright−Giemsa (Baso, Zhuhai, China) and identified as macrophages, eosinophils, neutrophils and lymphocytes based on cellular morphology and staining characteristics (**Supplementary Figure 2A**). At least 200 cells were counted under x400 magnification.

### 2.4 Histopathological analysis

After BALF was collected, the left lung tissues were completely removed, then fixed with 4% paraformaldehyde, embedded, processed into 4μm sections and stained with hematoxylin and eosin (HE) and periodic acid-Schiff (PAS). The infiltration of inflammatory cells and the level of mucus secretion in the airway was scored as previously described (19).

### 2.5 Airway resistance and dynamic compliance

Pulmonary function was carried out by the AniRes2005 animal lung function analysis system (Bestlab High-Tech, Beijing, China). After system calibration, all mice were anesthetized with pentobarbital sodium, tracheostomized, and placed in a forced pulmonary maneuver system. Mice were administered methacholine chloride (Mch, Sigma-Aldrich, USA) with doses increasing in multiples via the jugular vein. RI and Cdyn were measured.

### 2.6 Measurement of cytokines, double-stranded DNA and myeloperoxidase-DNA in BALF

Concentrations of interleukin IL6 and IL8 (Mouse KC) in BALF were quantitated by enzyme-linked immunosorbent assay (ELISA) kit (YOBIBIO, Shanghai, China) according to the manufacturers’ instructions. The amount of dsDNA in the BALF was evaluated according to the instructions of the kit (Thermo Fisher Scientific, USA). The MPO-DNA complex was used as a quantified marker of NET release with a capture ELISA (YOBIBIO, Shanghai, China).

### 2.7 Immunofluorescence staining of NETs in lung section

To identify NETs from lung tissues, the samples were first undergone antigen retrieval (citrate buffer, pH 6.0), blocking (5% BSA/0.3% Triton X-100), and then incubation with anti-citrullinated histone H3 (CitH3, 1:200, Abcam, ab5103) and anti-myeloperoxidase (MPO, 1:200, Abcam, ab300650) antibodies at 4°C overnight before treatment with secondary antibodies. After 4’,6-Diamidino-2-phenylindole (DAPI) staining, the sections were observed by a Leica SP8 confocal system, and images were captured randomly by independent experimenters blinded to group allocation throughout the study.

### 2.8 Extraction of neutrophils from bone marrow

Mice neutrophils were isolated from bone marrow. Briefly, the femurs and tibias were harvested and stripped of all muscle and sinew. The bone marrow was flushed out with 10 mL of RPMI medium containing 5% fetal bovine serum and 2mM EDTA on ice, then filtered (100um), and subjected to discontinuous Percoll gradient centrifugation (52%,65%,78%) at 500×g (30min, 4°C). The cells were gently aspirated from the neutrophil layer (65-78%), followed by deleting the erythrocytes and resuspension in Hanks’ Balanced Salt Solution (HBSS) containing 1% fetal bovine serum. HE and trypan blue staining was performed to identify the proportion and viability of neutrophils.

### 2.9 RNA sequencing and transcriptomic analysis of neutrophils

Neutrophil RNA was extracted with TRIzol (Invitrogen, USA). BGI DNBSEQ performed 150-bp paired-end sequencing. Low-quality reads (Q<15), adapters and poly-N were filtered. HISAT2 (v2.2.1) aligned clean reads to GRCm39, HTseq (v0.6.0) generated counts. DEGs were identified using the DEGseq method and screened with the criteria of Q value ≤ 0.05 and|log2FC|≥ 1. Kyoto Encyclopedia of Genes and Genomes (KEGG) and Gene Ontology (GO) enrichment analysis was also carried out with the online platform Dr. Tom (BGI Company, Shenzhen, China).

### 2.10 Statistical analysis

All statistics and graphs were performed using GraphPad Prism software v9.4.1 (San Diego, CA, USA). One-way ANOVA with Tukey’s *post hoc* method was used for multiple comparisons. Data are presented as means ± standard deviation (SD). Significant differences are shown as *P<0.05, **P<0.01, ***P<0.001, ****P<0.0001.

## 3 Results

### 3.1 The intratracheal administration of LPS exacerbated airway inflammation in OVA-induced asthmatic mice

**Figure 1:**
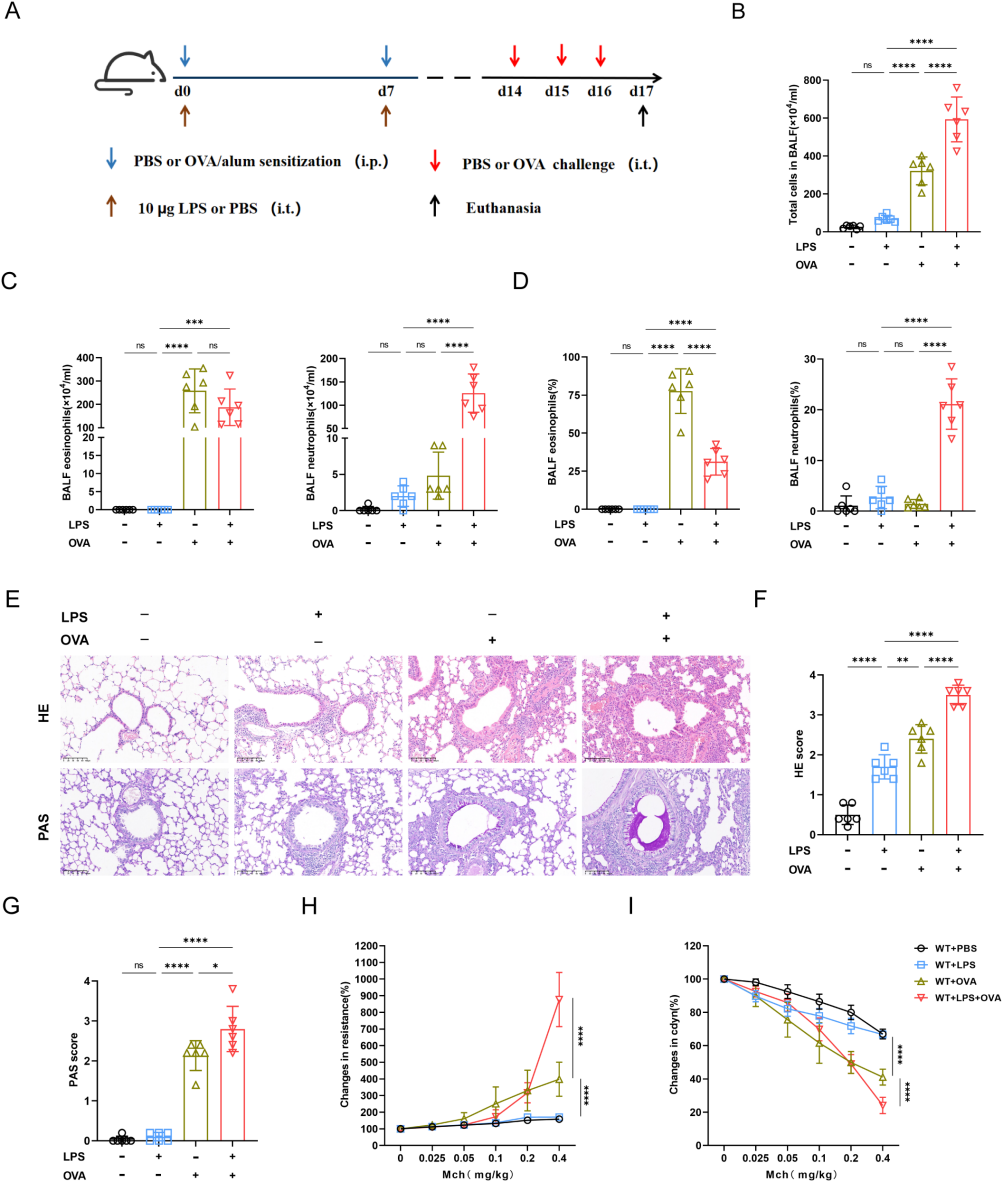
Combination of OVA and LPS produced the characteristics of neutrophil asthma in mice model. **(A)** Schematic of neutrophilic asthma induced by OVA combined with LPS. During the sensitization stage, 100ug OVA was injected intraperitoneally and 10ug of LPS was instilled intratracheally, then 25ug OVA was instilled intratracheally for challenging from day 14 to day 16. **(B)** The number of total cell counts was increased significantly in OVA+LPS group. **(C)** The statistical analysis of the eosinophil and neutrophil count. **(D)** The statistical analysis of the proportions of eosinophils and neutrophils. **(E)** Hematoxylin-Eosin (HE) staining and Paraffin acid-Schiff (PAS) staining of lung tissue (scale bar=100μm). **(F)** Histopathological score of HE staining. **(G)** Quantification of mucus-producing goblet cells of the PAS staining. **(H)** Measurement of airway resistance in mice undergoing methacholine challenge. **(I)** Lung dynamic compliance was measured in each group. Data were shown as mean ± SD, n=6. Significance between groups was calculated using one-way ANOVA with Tukey’s *post hoc* method. *p<0.05, **p < 0.01, ***p<0.001 and ****p<0.0001. ns: not significant, P>0.05.

To establish the mouse model of neutrophilic asthma, adult C57BL/6J mice were intraperitoneally (i.p.) sensitized with ovalbumin–aluminum hydroxide (OVA/alum) on days 0 and 7, and then challenged with OVA intratracheally (i.t.) on days 14, 15 and 16. LPS was instilled intratracheally (i.t.) during the sensitization phase (**Figure 1A**).

Compared with OVA group, the combination of LPS and OVA significantly increased the airway total cell counts, neutrophil count and proportion (**Figures 1B-D**), indicating that neutrophils were accumulating in airway. Intriguingly, despite the LPS+OVA group exhibiting a significantly lower proportion of eosinophil compared to the OVA group, their eosinophil counts were similar (**Figures 1C, D**). There was no notable difference in BALF cell count or neutrophil proportion between LPS and PBS groups. However, total cells, neutrophil counts and proportions in BALF of LPS group significantly increased by day 8 (**Supplementary Figures 3A-D**), suggesting that under non-allergic conditions, airway neutrophils had disappeared by day 17. Both HE and PAS scores were significantly elevated in LPS+OVA group. Compared with other groups, LPS group did not exhibited airway hypersecretion, indicating that LPS alone was insufficient to induce persistent mucin secretion (**Figures 1E-G**). Both airway resistance and lung dynamic compliance were more compromised in LPS+OVA group than in OVA group (**Figures 1H, I**). However, LPS intervention alone did not result in airway hyperreactivity.

Taken together, the LPS+OVA mouse model demonstrated features of neutrophilic asthma, including airway inflammation, hypersecretion, hyperresponsiveness, and significant infiltration of neutrophils.

### 3.2 Neutrophils contributed to the exacerbation of asthma induced by both LPS and OVA

**Figure 2:**
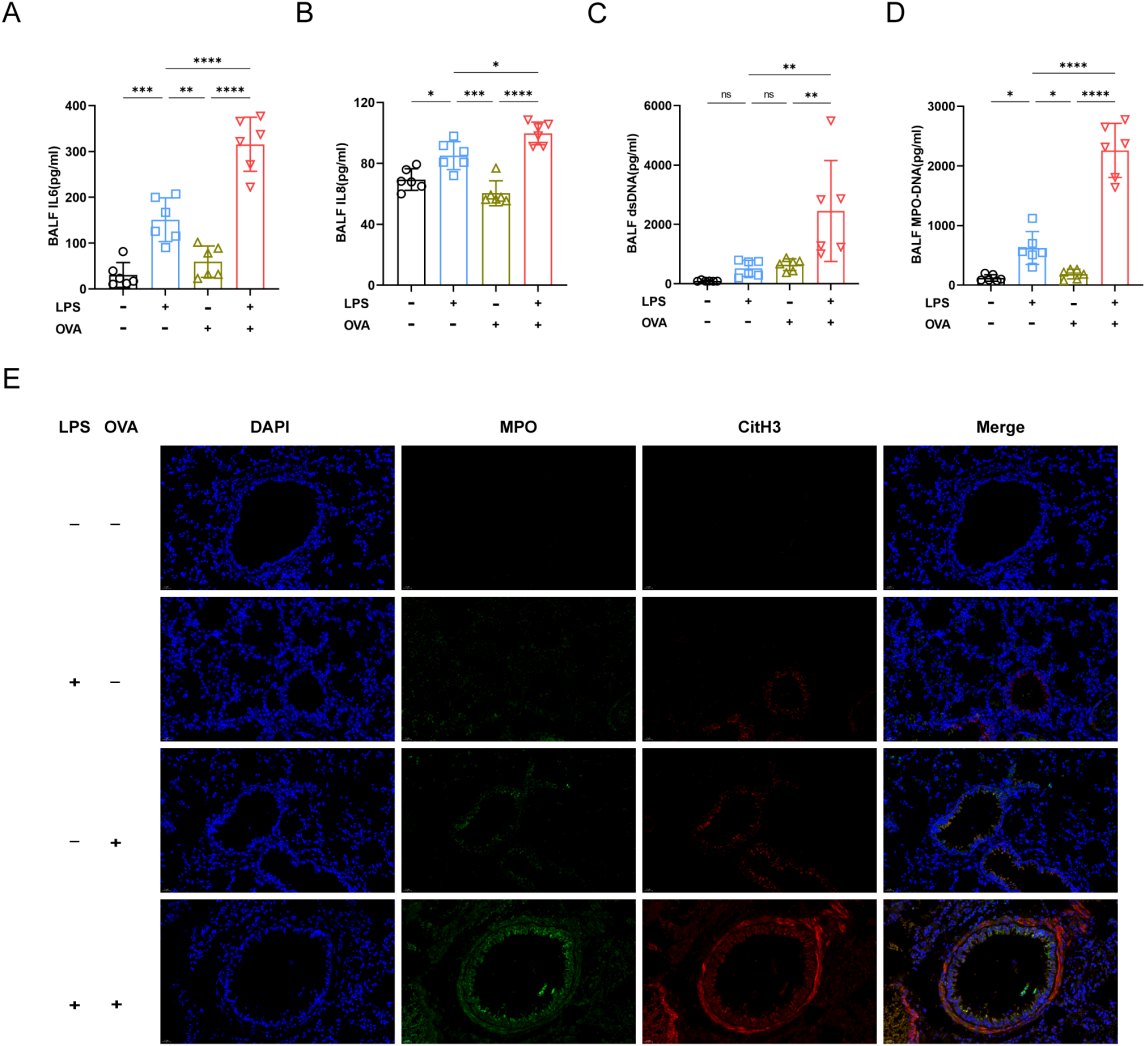
Neutrophil and NETs were involved in exacerbating asthma. **(A)** The concentration of IL6 in BALF was analyzed by ELISA. **(B)** Analysis of IL8 concentration in BALF. **(C)** The concentration of ds-DNA in BALF was analyzed. **(D)** MPO-DNA levels in BALF were measured. **(E)** Representative immunofluorescence images of NETs (Magnification, ×400), lung tissues were stained for myeloperoxidase (MPO, green), citrullinated histone 3 (CitH3, red) and DAPI (nuclear staining, blue). Data were shown as mean ± SD, n=6. Significance between groups was calculated using one-way ANOVA with Tukey’s *post hoc* method. *p<0.05, **p<0.01, ***p<0.001 and ****p<0.0001. ns: not significant, P>0.05.

Previous studies reported that IL6 and IL8 were associated with neutrophil recruitment (19, 20). Our findings showed elevated IL6 and IL8 levels in LPS+OVA group, aligning with increased airway neutrophil infiltration. (**Figures 2A, B**). The dsDNA concentration in the BALF of LPS+OVA group was much higher than in other groups, with no significant differences among the PBS, LPS, and OVA groups (**Figure 2C**). On day 8, dsDNA levels rose dramatically in LPS and LPS + OVA groups, consistent with BALF neutrophil counts (**Supplementary Figure 3E**). Given that dsDNA may originate from various cellular sources, we quantified myeloperoxidase-DNA (MPO-DNA). Results indicated that LPS treatment significantly elevated MPO-DNA levels in BALF, particularly when combined with OVA (**Figure 2D**).

Immunofluorescence staining of lung tissue sections was next conducted. As anticipated, in LPS+OVA group, there was a substantial co-localization of MPO and citrullinated histone H3 (CitH3), indicating the release of NETs by lung neutrophils (**Figure 2E**).

### 3.3 Suppression of neutrophil generation mitigated the airway inflammation induced by the combined LPS and OVA treatment

**Figure 3:**
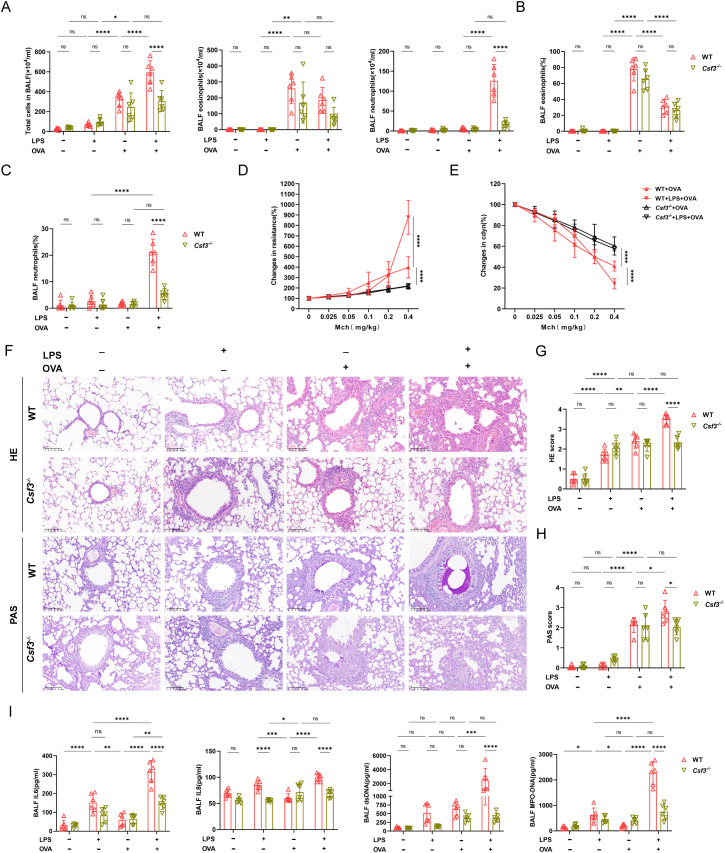
Deficiency in neutrophil production attenuated airway inflammation in neutrophilic asthma. **(A)** BALF cell counts were measured of WT mice and *Csf3^-/-^* mice. **(B)** The proportion of eosinophils in two kinds of mice was determined. **(C)** The proportion of neutrophils was analyzed. **(D)** Analysis of airway resistance in two kinds of mice undergoing methacholine challenge. **(E)** Lung dynamic compliance was measured in each group. **(F)** Representative images of HE staining and PAS staining of lung tissue (scale bar=100μm). **(G)** Inflammatory scores of HE staining of Lung sections. **(H)** Percentage of PAS staining goblet cells. **(I)** The levels of IL6, IL8, ds-DNA, MPO-DNA in BALF. Data were shown as mean ± SD, n=6. Significance between groups was calculated using one-way ANOVA with Tukey’s *post hoc* method. *p<0.05, **p<0.01, ** *p<0.001 and ****p<0.0001. ns: not significant, P>0.05.

**Figure 4:**
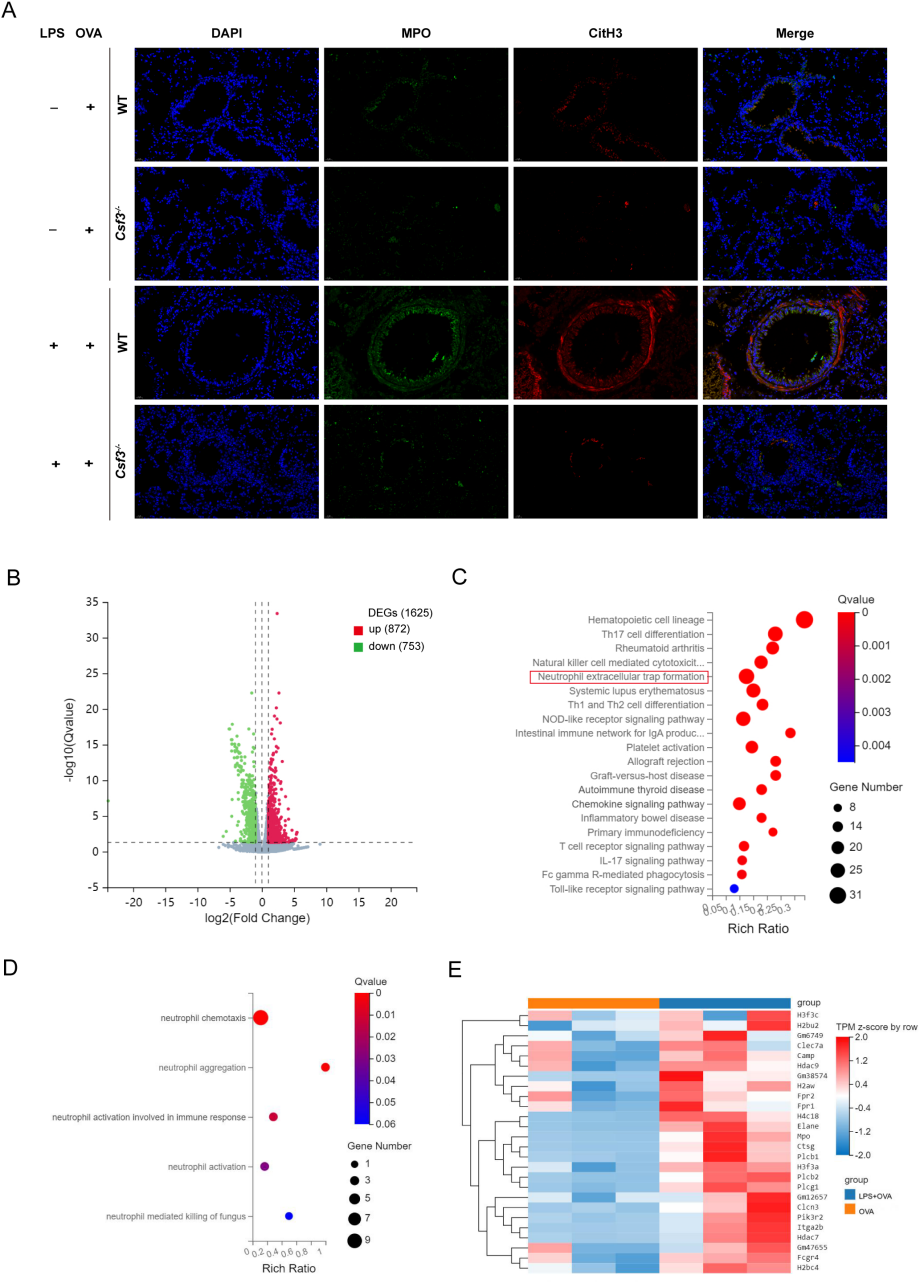
NETs contributed to the exacerbation of asthma induced by LPS. **(A)** Representative immunofluorescence images of NETs (Magnification, ×400), lung sections were stained for myeloperoxidase (MPO, green), citrullinated histone 3 (CitH3, red) and DAPI (nuclear staining, blue). **(B)** Volcano plot for differential gene expression between WT+OVA group and WT+LPS+OVA group. **(C)** Bubble plot of KEGG enrichment analysis of differential genes involved in immune pathway. **(D)** Bubble plot of GO enrichment analysis of differential genes involved in immune pathway. **(E)** Heat map for differential gene expression involved in NETs. n=6 in **(A)**, n=3 in **(B-E)**.

To explore the role of neutrophils in neutrophilic asthma, we conducted experiments using colony-stimulating factor3 (*Csf3*) deficient mice with significantly reduced neutrophils *in vivo*. In *Csf3* ^-/-^mice, OVA could induce allergic asthma characterized by eosinophilic airway inflammation and airway hyperresponsiveness (**Figures 3A-H**). In LPS+OVA group, *Csf3* ^-/-^ mice had significantly lower total cell, neutrophil counts and neutrophil proportion in BALF compared to WT mice, while eosinophil levels were similar in both groups (**Figures 3A-C**). Additionally, the pulmonary resistance in *Csf3* ^-/-^ mice in the LPS+OVA group was significantly lower than that in WT mice and even lower than in the OVA group of WT mice. Notably, it was comparable to the OVA group in *Csf3* ^-/-^ mice (**Figure 3D**). The lung compliance results mirrored those of lung resistance (**Figure 3E**), indicating that neutrophils may play a crucial role in the development of airway hyperresponsiveness. Similar to WT mice, LPS intervention did not exacerbate lung function (**Supplementary Figures 4A, C**). In LPS+OVA group, *Csf3* ^-/-^ mice exhibited reduced levels of airway inflammatory infiltration and mucus hypersecretion compared to WT mice (**Figures 3F-H**). The neutrophil related inflammatory indicators such as IL6, IL8 and dsDNA, MPO-DNA which represented levels of NETs were analyzed, similar results were obtained in LPS+OVA group between *Csf3* ^-/-^ mice and WT mice (**Figure 3I**). Immunofluorescence staining conducted on lung tissues demonstrated a significant reduction in NETs co-localizing with MPO and citH3 in LPS+OVA group of *Csf3* ^-/-^ mice (**Figure 4A**).

### 3.4 NETs were involved in the mechanism of asthma exacerbated by LPS at the transcriptome level

To further investigate the mechanism by which neutrophils contribute to asthma exacerbation, we isolated bone marrow-derived neutrophils from the OVA and LPS+OVA groups of WT mice and performed transcriptome sequencing analysis. This analysis identified 1,625 DEGs, with 872 genes up-regulated and 753 genes down-regulated (**Figure 4B**; **Supplementary Figure 5A**). Among these, 184 DEGs were related to immune function. We conducted Kyoto Encyclopedia of Genes and Genomes (KEGG) and Gene Ontology (GO) analyses on the DEGs involved in immune pathways. The KEGG analysis indicated significant enrichment of DEGs in the neutrophil extracellular trap formation pathway (**Figure 4C**). GO analysis revealed that DEGs were significantly enriched in processes related to neutrophil chemotaxis, aggregation, and activation (**Figure 4D**). A heatmap was generated to visualize the expression levels of 26 DEGs and 179 genes associated with the NETs pathway (**Figure 4E**; **Supplementary Figure 5B**). It was observed that, compared to the OVA group, the DEGs and genes in LPS+OVA group exhibited significantly higher expression levels. These findings suggested that neutrophils and NETs may be involved in neutrophilic asthma at the transcriptome level. Interestingly, KEGG pathway analysis of immune-related DEGs in LPS group versus PBS group did not show enrichment in the NETs pathway (**Supplementary Figures 5C, D**), implying that under allergic conditions, neutrophils and their NETosis function may be activated for a prolonged period.

### 3.5 Impairments in NETs formation significantly alleviated the exacerbation of asthma induced by LPS

**Figure 5:**
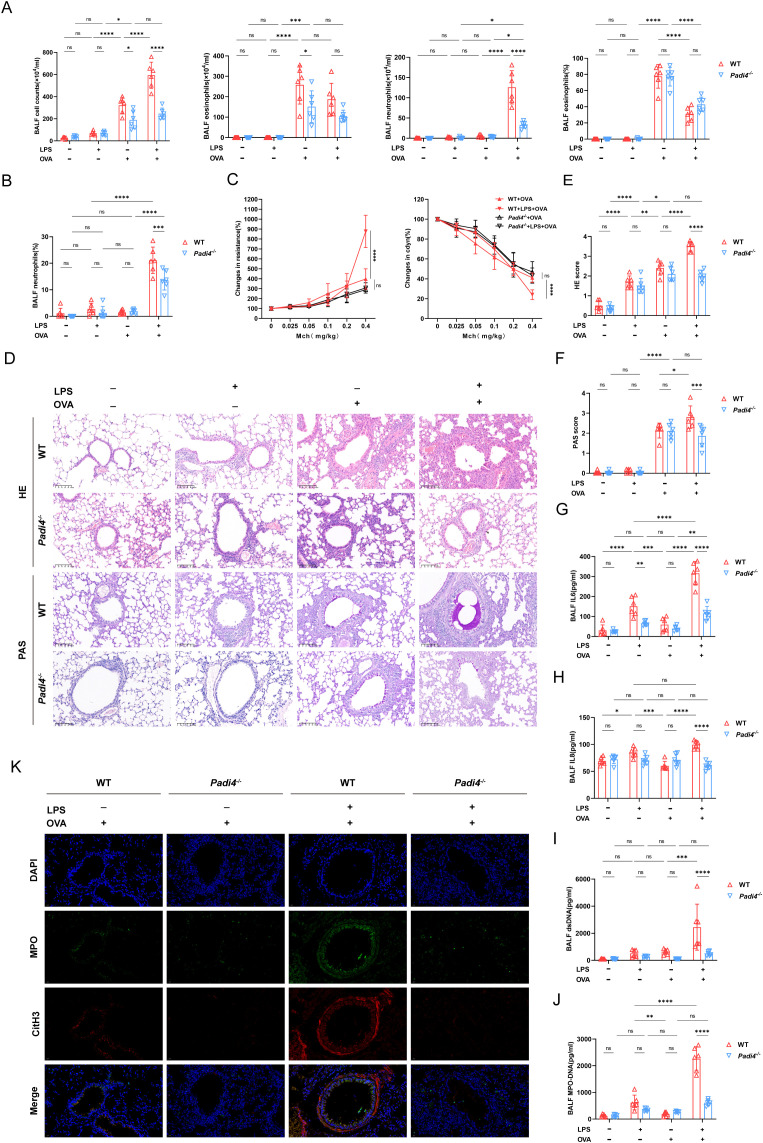
Deficiency in Neutrophil extracellular traps production alleviated asthma features. **(A)** Total cell counts, eosinophils, and neutrophils count and percentage of eosinophils in BALF was calculated in WT mice and *Padi4^-/-^* mice. **(B)** The proportion of neutrophils was evaluated. **(C)** Airway resistance and lung dynamic compliance was analyzed in each group. **(D)** Representative histological images of HE and PAS staining of lung, scale bar=100μm. **(E)** Histopathological score of HE staining of lung Sections. **(F)** Histopathological score of PAS staining of lung sections. **(G)** The level of IL6 in BALF detected with ELISA. **(H)** The level of IL8 in BALF detected with ELISA. **(I)** The concentration of ds-DNA in BALF was detected. **(J)** The concentration of MPO-DNA was measured in BALF. **(K)** Representative immunofluorescence pictures of NETs (Magnification, ×400), lung sections were stained for myeloperoxidase (MPO, green), citrullinated histone 3 (CitH3, red) and DAPI (nuclear staining, blue). Data were shown as mean ± SD, n=6. Significance between groups was calculated using one-way ANOVA with Tukey’s *post hoc* method. *p<0.05, **p<0.01, ***p<0.001 and ****p<0.0001. ns: not significant, P>0.05.

To further explore the role of NETs in neutrophilic airway inflammation, we conducted experiments utilizing peptidyl arginine deiminase 4 (*Padi4*) deficient mice. *Padi4* participated in the formation of NETs by catalyzing the citrullination of arginine residues in histones. Knocking out the *Padi4* gene significantly inhibited the generation of NETs. In *Padi4* ^-/-^mice, we established a conventional model of allergic asthma via OVA sensitization and challenge, characterized by inflammatory cell infiltration, goblet cell hypersecretion, and airway hyperresponsiveness. Treatment with OVA in conjunction with LPS led to an increase in neutrophil count and proportion, but did not exacerbate the aforementioned asthma-related parameters (**Figures 5A-F**). Compared to the WT+LPS+OVA group, the total cell count, neutrophil count, and proportion in the BALF of the *Padi4* ^-/-^+LPS+OVA group were significantly reduced. However, there was no significant difference in the proportion and count of eosinophils (**Figure 5A**). In Padi4^-/-^ mice, lung resistance and dynamic compliance in LPS+OVA group were also significantly improved compared to those in WT mice (**Figure 5C**). Similar to WT and *Csf3^-/-^* mice, LPS intervention did not exacerbate lung function (**Supplementary Figures 4B, D**). Surprisingly, the lung resistance in LPS+ OVA group of *Padi4* ^-/-^ mice was significantly higher than that of *Csf3* ^-/-^mice (**Supplementary Figure 4E**), suggesting that, beyond NETs, additional neutrophil functions may contribute to airway hyperresponsiveness. As expected, Inhibiting NETs formation improved inflammatory infiltration and mucus hypersecretion (**Figures 5D-F**). After knocking out *Padi4*, the combination of LPS and OVA did not exacerbate the production of IL6 and IL8 (**Figures 5G, H**), nor did it increase the production of dsDNA and MPO-DNA (**Figures 5I, J**). The co-localized MPO-CitH3 immunofluorescence further confirmed a significant reduction of NETs generation in LPS+OVA group (**Figure 5K**). In other groups, there were no significant differences among mice with different genotypes (**Supplementary Figure 2B**).

## 4 Discussion

Excessive airway neutrophilia is reported in 20%–30% of asthmatic patients (8). Increasing studies have shown that neutrophils infiltrating the airway may be involved in airway inflammation and damage, especially in severe asthma (21, 22). In this study, LPS combined with OVA was used to establish a neutrophilic asthma model. After neutrophil production was impaired, the airway inflammation was significantly alleviated. Transcriptome analysis of bone marrow neutrophils from WT mice revealed that the DEGs were enriched in the NETs pathway. Similar results were obtained when NETs generation was inhibited.

LPS is frequently utilized in conjunction with OVA or HDM to develop mice models of neutrophilic airway inflammation (3, 10, 23). In contrast to the administration of LPS during the challenge phase (23, 24). we intratracheally instilled LPS into mice during the sensitization phase to mitigate confounding effects from acute lung injury. It has been observed that variations in LPS dosage, timing of intervention, and the developmental stage of experimental subjects yielded disparate outcomes (10, 18, 19, 25). explaining the contradiction between the exacerbation of asthma by LPS in reality and the hygiene hypothesis.

LPS is capable of directly activating the classical complement pathway through its lipid A domain, independent of antigen-antibody complexes. Furthermore, LPS may also trigger the complement cascade via both the lectin and alternative pathways (4, 26). In murine models of LPS-induced sepsis and OVA-sensitized allergic asthma, pulmonary expression levels of the complement anaphylatoxin receptors C3aR, C5aR1, and C5aR2 were markedly elevated (27). Locally activated complement components (e.g., C3a and C5a) trigger a cascade of inflammatory injury by recruiting neutrophils and other immune cells to lung tissue via chemotaxis (4–6), thereby mediating tissue damage and small airway dysfunction, as well as potential airway tissue remodeling (5, 28, 29). The precise mechanistic cascade of complement activation in our LPS exacerbated murine model of asthma remains to be fully elucidated and warrants systematic investigation in future studies.

Neutrophils are characterized by a relatively brief lifespan. However, their survival is notably prolonged in a localized inflammatory microenvironment (12, 30). This study found that neutrophils recruited into the airway via LPS intervention alone dissipated rapidly. Conversely, in allergic conditions, these neutrophils exhibited extended survival. Research by Thomas et al. demonstrated that locally instructed CXCR4^hi^ neutrophils contributed to the exacerbation of allergic airway inflammation. This finding underscores the heterogeneity, plasticity, and adaptability of neutrophils in specific environments (17). Inflammatory cytokines IL-6 and IL-8, produced by airway epithelial cells, macrophages, and neutrophils themselves, promote neutrophil recruitment and activation (14, 19, 20). When we reduce the generation of neutrophils, the levels of both of them decrease significantly.

Granulocyte colony-stimulating factor (G-CSF) orchestrates granulopoiesis through multi-tiered regulatory mechanisms. Primarily, it induces C/EBPβ and PU.1 expression to direct granulocytic lineage commitment. Furthermore, G-CSF mobilizes neutrophils into peripheral circulation, a process critical for emergency granulopoiesis (12). A recent study found that using anti-ly6G to remove neutrophils unexpectedly increased G-CSF levels, which then activated type 2 innate lymphoid cells (ILC2) to enhance type 2 airway inflammation (31). The discrepancy in our experiment likely stems from our methodology, which involved knocking out the CSF gene to prevent G-CSF level increases. Although reducing neutrophils can decrease airway inflammation, it may also weaken immune defense, making this approach unsuitable for clinical use. NETs are intricate networks of chromatin and proteins released from neutrophils during the programmed cell death known as NETosis. A growing body of studies have shown that NETs are not only present in the sputum and serum of asthma patients, but also closely related to the severity of asthma (32, 33). This study observed increased dsDNA and MPO-DNA in BALF of mice with neutrophilic asthma, indicating a potential association between NETs and airway inflammation. Transcriptomic analysis of bone marrow neutrophils in WT mice showed DEGs mainly enriched in NET-related pathways, aligning with previous research (17). These observations suggest that targeted inhibition of NETs could be a viable strategy for mitigating airway neutrophilic inflammation.

*Padi4* is presently considered to facilitate NETosis through the citrullination of histones, which leads to chromatin decondensation by reducing the electrostatic interactions between histones and DNA. Neutrophils derived from Padi4 knockout mice do not exhibit citrullinated histone H3 and fail to undergo NETosis when stimulated by LPS (34). This study found that inhibiting NETs reduced airway inflammation, decreased airway neutrophils, and lowered IL-6, IL-8, dsDNA and MPO-DNA levels. In chronic airway inflammatory diseases, NETs often exhibit a dual role. While they can provide immune defense by eliminating pathogens and degrading virulence factors, the accumulation of excessive NETs also exacerbate airway inflammation if not adequately degraded (35). Furthermore, NETs have been shown to intensify airway inflammation by enhancing the antigen-presenting capabilities of CD11b^+^Ly-6C^+^ dendritic cells (17). Histones in NETs could enhance Th17 cell differentiation, raising IL-17 levels and worsening neutrophilic inflammation (36). NETs was negatively correlated with FEV1% predicted (37). Extensive NETs formation in mucus plugs contributed to airway obstruction, further exacerbated the lung function (38). Interestingly, our study reveals that eliminating neutrophils improves lung function more notably than just inhibiting NETs, suggesting that neutrophils impair lung function through additional mechanisms beyond NETs. Neutrophils can cause airway narrowing by producing mediators like elastase or interacting with goblet cells, leading to mucus accumulation (39). Neutrophils also exacerbated the proliferation of airway smooth muscle cells by releasing extracellular vesicles and exosomes (22, 40).

Our study has limitations. Firstly, mice don’t naturally develop asthma like humans, but they are useful for early mechanism exploration due to their short experimental cycles, diverse genotypes, and available reagents. Secondly, *Padi4* deficiency might also lower extracellular DNA release from other immune cells, such as Eosinophil extracellular traps, thus further reducing airway inflammation. Finally, bone marrow neutrophils were used for transcriptome sequencing due to technical constraints. Considering the plasticity of neutrophils, in the future, lung single-cell sequencing can be considered to further verify our results.

In summary, delayed apoptosis of neutrophils and excessive NETs in lung exacerbate airway inflammation and impair lung function in asthmatic mice aggravated by LPS. Therefore, targeted inhibition of NETs formation may offer therapeutic potential in mitigating neutrophil-driven airway inflammation.

## Data Availability

The datasets generated during and/or analyzed during the current study are available from the corresponding author on reasonable request.
