# Supplementary material for: The role of neutrophils and NETosis in lipopolysaccharide exacerbated asthmatic airway inflammation
Source: Front Immunol. 2025 Sep 2;16:1651085. doi: 10.3389/fimmu.2025.1651085 (PMC12436110; doi:10.3389/fimmu.2025.1651085)
Supplement: Supplementary file 1 [file DataSheet1.docx]

Supplementary Material

## Supplementary Figures

**
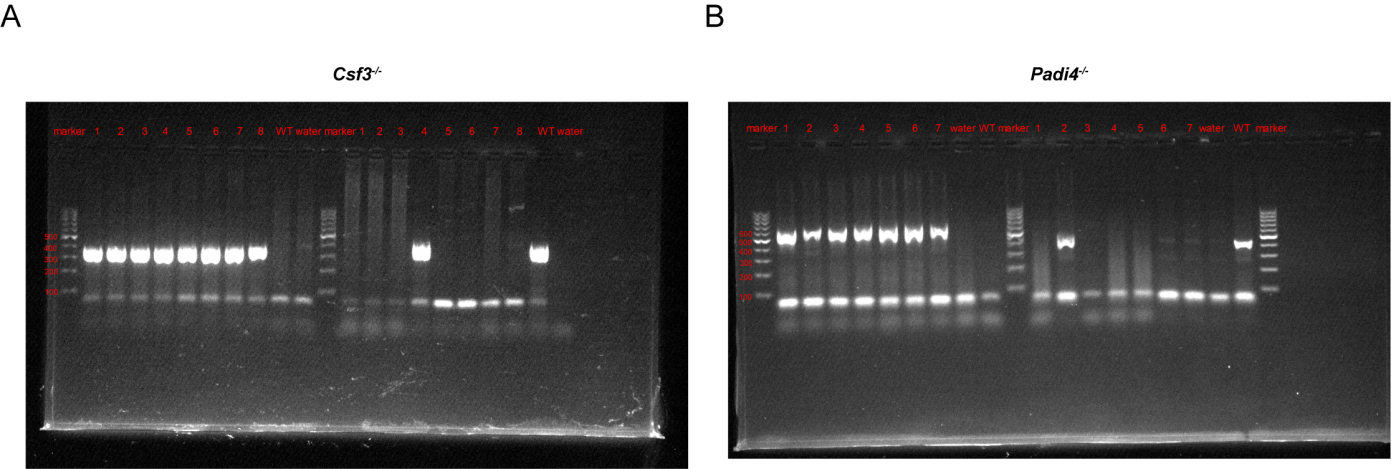
**

**Supplementary Figure 1 Genotyping analysis of mouse tails. A** Agarose gel electrophoresis of the PCR products from *Csf3*^-/-^ mouse tails. In *Csf3*^-/-^ homozygous mice, there is only one band for Primer 1 on the left side. **B** Agarose gel electrophoresis of the PCR products from *Padi4*^-/-^ mouse tails. In *Padi4*^-/-^ homozygous mice, there is only one band for Primer 1 on the left side. Csf3, Colony-stimulating factor 3; Padi4, Peptidyl arginine deiminase 4.

**
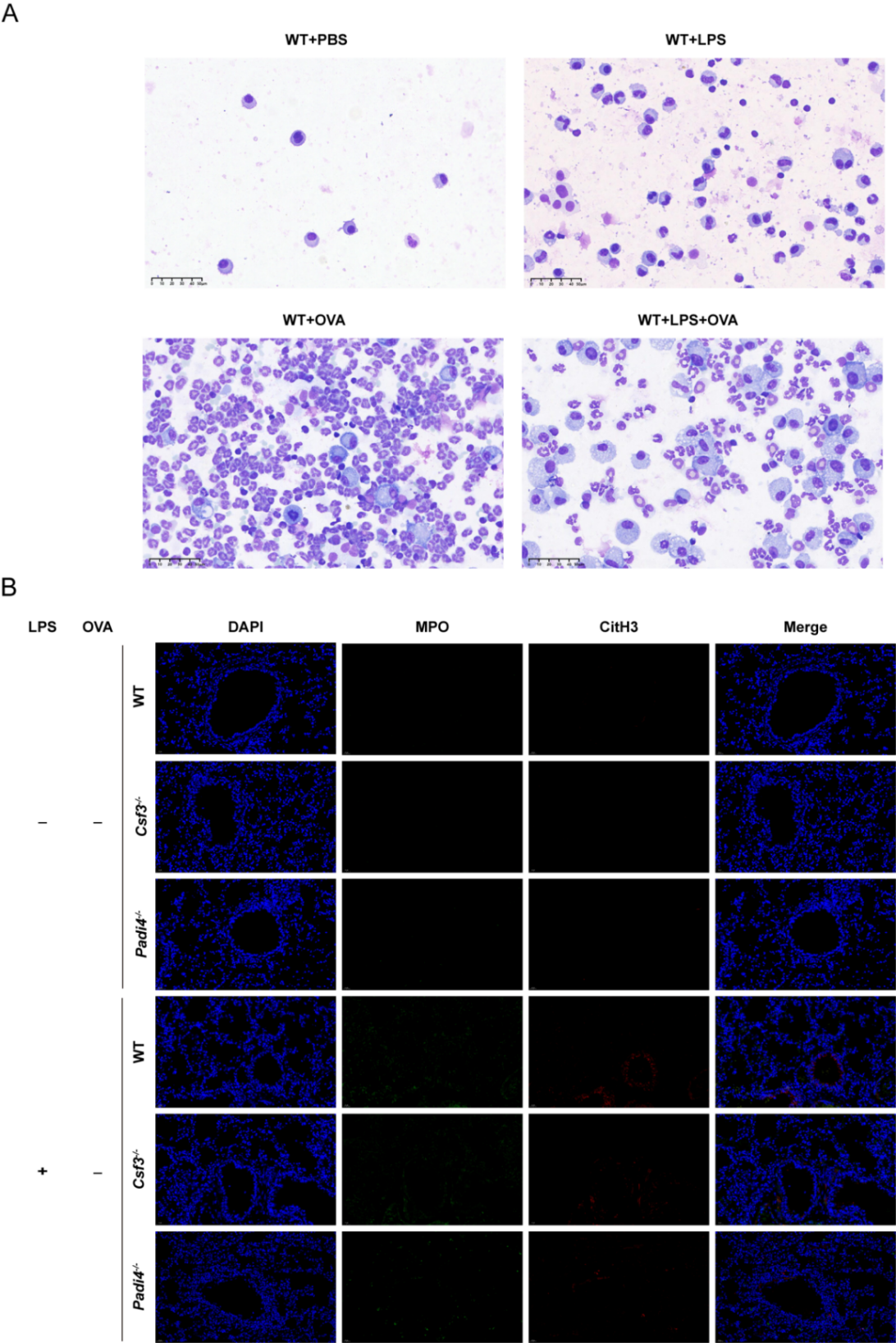
**

**Supplementary Figure 2 Images of cell and tissue staining. A** Representative images of BALF cells stained with Wright‑Giemsa (Magnification, ×400). **B** Representative immunofluorescence pictures of NETs (Magnification, ×400) in each group under PBS or LPS intervention.

**
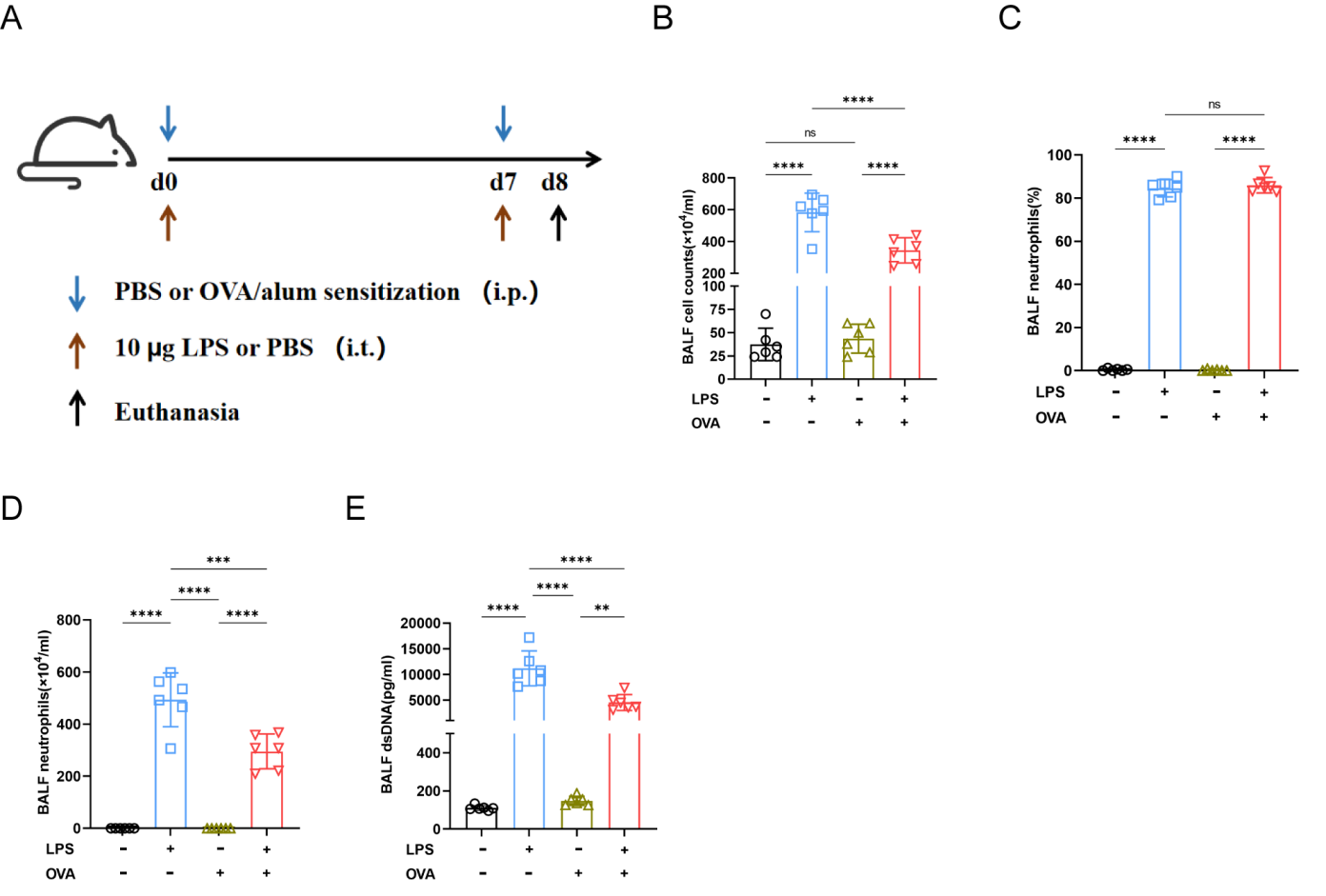
**

**Supplementary Figure 3 Neutrophils and ds-DNA levels in BALF of WT mice on day 8. A** Mouse model experimental schemes. **B** Total cell count in BALF was calculated. 8. **C** The proportion of neutrophils in BALF was analyzed. **D** Neutrophil count in BALF of WT mice on day 8. **E** The concentration of ds-DNA in BALF was detected. Data were shown as mean ± SD, n=6. Significance between groups was calculated using one-way ANOVA with Tukey’s post hoc method. *p<0.05, **p<0.01, ***p<0.001 and ****p<0.0001.


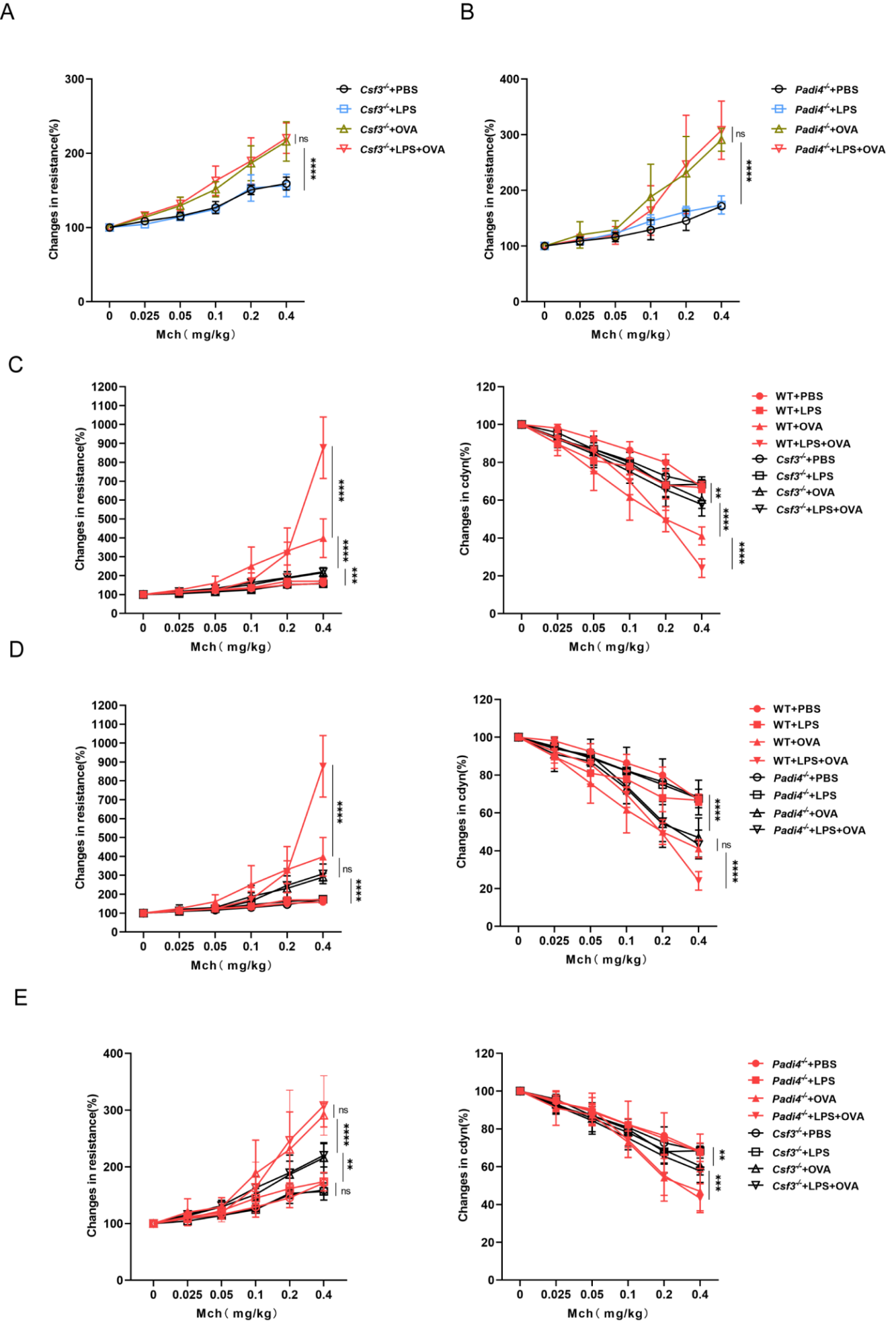


**Supplementary Figure 4 Airway resistance and lung dynamic compliance was analyzed in each group. A** Airway resistance was measured in *Csf3*^-/-^ mice. **B** Airway resistance was analyzed in *Padi4*^-/-^ mice. **C** Airway resistance and lung dynamic compliance was analyzed in WT and *Csf3*^-/-^ group. **D** Lung function was analyzed in WT and *Padi4*^-/-^ group. **E** Airway resistance and lung dynamic compliance was analyzed in *Csf3*^-/-^ and *Padi4*^-/-^ group. Data were shown as mean ± SD, n=6. Significance between groups was calculated using one-way ANOVA with Tukey’s post hoc method. *p<0.05, **p<0.01, ***p<0.001 and ****p<0.0001.


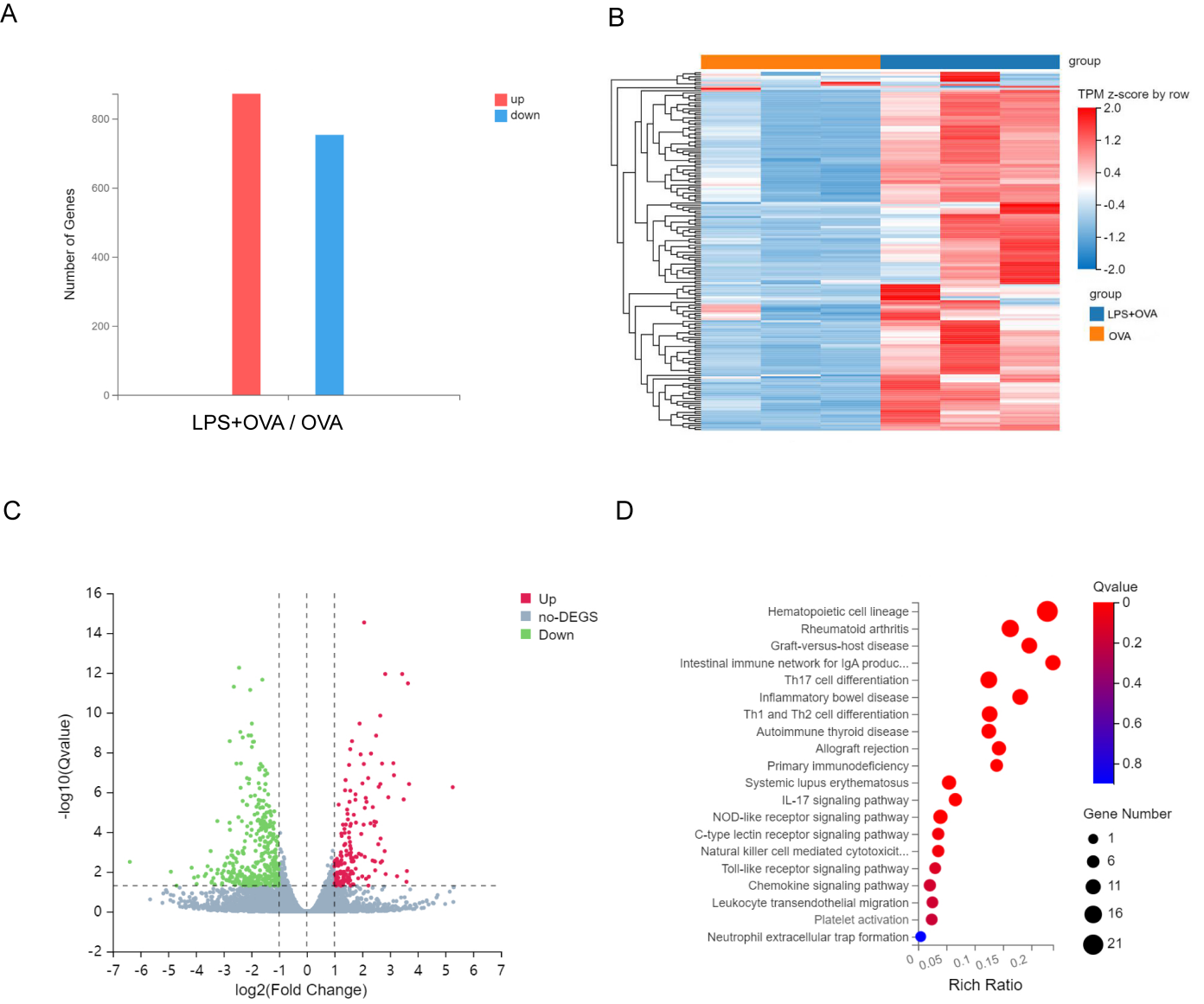


**Supplementary Figure 5 RNA sequencing and transcriptomic analysis of neutrophils. A** Bar chart of differentially expressed genes between LPS+OVA group and OVA group. **B** Heat map of genes involved in the NETs pathway. **C** Volcano plot for differential gene expression between WT+PBS group and WT+LPS group. **D** Bubble plot of KEGG enrichment analysis of differential genes involved in immune pathway. n=3.
